# COVID-19 Passport as a Factor Determining the Success of National Vaccination Campaigns: Does It Work? The Case of Lithuania vs. Poland

**DOI:** 10.3390/vaccines9121498

**Published:** 2021-12-18

**Authors:** Marcin Piotr Walkowiak, Justyna B. Walkowiak, Dariusz Walkowiak

**Affiliations:** 1 Department of Preventive Medicine, Poznan University of Medical Sciences, 60-781 Poznań, Poland; MarcinWalkowiak@wir.pl; 2 Department of Language Policy and Minority Studies, Adam Mickiewicz University in Poznan, 61-712 Poznań, Poland; justwalk@amu.edu.pl; 3 Department of Organization and Management in Health Care, Poznan University of Medical Sciences, Przybyszewskiego 39, 60-356 Poznań, Poland

**Keywords:** COVID-19, vaccination, vaccination coverage, trust in vaccines, interventions to increase vaccination coverage, public health

## Abstract

As the ongoing COVID-19 pandemic poses a global threat, it is of utmost importance that governments should find effective means of combating vaccine hesitancy and encouraging their citizens to vaccinate. In our article, we compare the vaccination outcomes in the past months in two neighbouring post-communist EU states, Lithuania and Poland. Both introduced COVID-19 certificates, but only the former followed with gradual limitations for those who failed to get vaccinated, beginning with restricted access to restaurants, sports facilities and indoor events, and finally banning residents without a certificate from entering supermarkets or larger shops and using most services. By contrast, in Poland, the certificate remained a tool for international travel only. We show using statistical data that Lithuania’s strict policy, regardless of its social implications, led to markedly higher vaccination outcomes in all age groups than those in Poland at the time.

## 1. Introduction

The much-debated and at the same time highly contentious issue of the effectiveness of COVID-19 certificates in their domestic use in European countries is especially timely in the last quarter of the year 2021, as national governments are struggling with a surge in new cases of people tested positive, while vocal anti-vaccine movements take effect in discouraging wide segments of the population from getting vaccinated. In our study, we decided to focus on the comparison of the vaccination outcomes in two EU states: Lithuania and Poland. They have a common border and common history, having functioned as one polity from the late 14th c. to the close of the 18th, when they both simultaneously lost statehood to stronger neighbours and afterwards jointly repeatedly fought for independence. To this day there is a Polish minority in Lithuania and a Lithuanian minority in Poland. Moreover, both countries share communist and post-communist history [1,2,3,4], comparable COVID-19-related morbidity [4,5], and proportion of the vaccines used in the W27–W43 period (Pfizer 58.5% for Lithuania and 56.3% for Poland, Janssen 34.9% and 42.8% respectively, Moderna 5.5% and 0.6%, AstraZeneca 1.1% and 0.3%), as well as originally very similar vaccination outcomes. However, the decision of the Lithuanian government in August 2021 to introduce, since September 13, new rules for the functioning of the so-called opportunity passport (*galimybių pasas*) enabled us to gain a unique insight into the effectiveness of that measure and to isolate only one factor with potential impact on these outcomes.

The history of digital COVID-19 certificates in the EU is relatively short. The first stage in their development was the recommendation by the European Medicines Agency (EMA) of the first COVID-19 authorisation on 21 December 2020. The work of the European Parliament in the spring of 2021 resulted in the project receiving an official signature on June 14, coming into force on July 1 [6]. This initiative was especially timely in view of the negative results for the economies of EU member states, experienced in the aftermath of the first lockdown in the spring of 2020. The basis for their issuance was either the completion of the full vaccination cycle, or having recently recovered from the virus, or—in the absence of any of these—a recent negative test for COVID-19. These certificates, also called COVID-19 passports, health passes, green passes, safe tickets or corona passes, were to serve as a tool for both international travel and domestic access to services. While the former purpose—facilitation of safe free movement inside the EU—was covered by the EU law, it was left to the discretion of individual member states whether and to what extent they would use these documents to internally regulate access to cultural or sports events and facilities, indoor restaurants and cafés, transportation, or even workplaces in all or selected sectors [7].

Among the EU states, Italy was one of the first to use that opportunity to introduce restrictions domestically. Originally (since 6 August 2021) [8] they were obligatory in cinemas, restaurants, museums, gyms or swimming pools, as well as for school staff. However, according to the new law that took effect on October 15, the obligation to show a COVID pass was extended to all employees, who could face a fine of up to EUR 1500 for non-compliance [9]. The pass was even made mandatory for students at some Italian universities [10]. As of October 2021, over 80% of Italians aged 12 or more have been fully vaccinated, with the target level of 90% to be reached in December [11].

At the end of July 2021, the French parliament passed a law, effective August 1, making a health pass mandatory for access to restaurants, bars and some other public venues, as well as on domestic trains and planes. Earlier such a document had already been required in cultural and sports facilities accommodating over 50 people. Originally legally binding for adults only, since September 30 it was to take effect in relation to all citizens aged 12 or older. The new law also made provisions for obligatory vaccination of all healthcare workers, under threat of suspension. The effectiveness of the rather harsh service access restrictions in France was high [12]. The declaration by President Macron of restrictions in access to various services on July 12 led to a sharp increase in the number of residents getting vaccinated: the daily number of first-time vaccinations nearly doubled. In consequence, by October 14, a record-breaking, among the EU countries, 96% of the adult population of France had received at least one dose of vaccine.

By the end of July 2021, sixteen EU countries had decided to use COVID passports to restrict access to certain public spaces for their residents and visitors from abroad: Austria, Belgium, Cyprus, Denmark, France, Germany, Greece, Ireland, Italy, Latvia, Lithuania, Luxembourg, Netherlands, Portugal, Slovenia and Spain [13]. By 26 October 2021, more member states had followed suit: Bulgaria, Croatia, the Czech Republic, Estonia, Finland, Hungary and Slovakia. As of October 26, only three EU countries—Poland, Spain and Sweden—ultimately refrained from domestic use of the document, whereas Denmark, the first EU country to create such a document (*Coronapas* in Danish), later abolished in national law the domestic restrictions originally linked with it [10].

It must be noted that not only the timing of the COVID certificate but also the scope of restrictions for those without it may vary from country to country, which makes it difficult to effectively study and compare its effect. For instance, while Slovenia made a COVID certificate mandatory for all in situ employees [14], a COVID certificate in the Czech Republic has since 1 November 2021 restricted access only to restaurants, bars, nightclubs and other indoor places [15]. However, the effectiveness of the same measures adopted in particular countries may differ, due to different perceptions of the inconvenience they cause and to the consistency in their enforcement. Thus a COVID passport needed to enter a restaurant, for instance, will have more impact in a country with a tradition of frequently eating out: in 2019, the average inhabitant of Iceland spent EUR 4410 on restaurants and hotel stays, while the average Pole spent only EUR 310 [16].

Additionally, the technological solutions adopted in particular states tend to differ. Luxembourg, for example, developed its own application, called *CovidCheck*. In Estonia, the *Immuunsuspass*, the application tested as early as 2020, was ultimately replaced by the standard EU Digital COVID Certificate [17]. What is more, differences may reach the regional rather than state level: while Spain refrained from issuance of an all-country certificate, it was nevertheless planned for introduction by Galicia alone, though eventually those plans were thwarted [13].

A frequent phenomenon was the gradual extension of domestic access restrictions for those without a COVID certificate. A case in point is Latvia, where on 4 November 2021 the parliament passed a law that required all employees after November 15 to show a COVID certificate or face suspension, while earlier this obligation had applied only to workers in healthcare, education and social care [18]. Since 15 November 2021, Latvians with the certificate have had access to shops with shopping areas of over 1500 m^2^, adult education onsite and to church services, as well as to events of more than 500 participants. By contrast, those without the pass may only use small-size shops (with pharmacies, pet stores, hygiene stores, press vendors and gas stations excluded from restrictions), and they may visit religious sites only individually, for no longer than 15 min at a time [19]. The backdrop of tightened restrictions is the deteriorating pandemic situation in Latvia—the percentage of those vaccinated, 54% as of 31 October 2021, placed this country rather low in Europe—coupled with the fifth-lowest interest in getting vaccinated against COVID-19 in June–July 2021 among all the EU member states [20].

The idea for such a passport in Lithuania was first announced in Lithuanian media in April 2021 [21]. It was planned as an electronic document, developed independently of a similar EU project (Digital Green Certificate), and issued to every Lithuanian citizen over 16 years of age who either completed the full COVID-19 vaccination cycle, recovered from the COVID-19 infection with a positive test result obtained no more than 180 days before or tested COVID-negative within the past 24 h. The original provisions enabled its owner to fully enjoy a social life, which at the time (April–May) meant chiefly visiting restaurants, cafés, billiards clubs, escape rooms, bowling alleys, swimming pools, water parks and other sports facilities, as well as participating in concerts and similar cultural events. The document was eventually introduced on 24 May 2021. The main driving force behind this regulation, it seems, was the wish to support the national economy after some period of enforced stagnation by making it possible for restaurants or sports clubs to fully reopen and to organise cultural events.

On 9 July 2021, the President of the Lithuanian Business Confederation Andrius Romanovskis proposed in social media to give employers the right to ask employees for a COVID-19 certificate [22]. On July 13, Prime Minister Ingrida Šimonytė revealed in an interview [23] that as the epidemiological situation in Lithuania was deteriorating, more activities might become available only with the “opportunity passport”, especially considering that currently the slowest to vaccinate were the people aged 20–40, the most mobile and active group that should be the most interested in travelling and having fun. She did not rule out the government having “to use some negative incentives to politely remind citizens that vaccination has additional benefits”.

Ultimately, in the first days of August 2021, the media reported major changes to be made in the regulations concerning *galimybių pasas* [24]. Those who did not have one were from that time on prevented from using many more services than before, including those of hairdressers, beauty parlours, travel agents or repair shops, as well as from entering supermarkets and larger shops. When the new rules took effect on September 13, the remainder of the population thus effectively lost access to many popular services and commodities. As regards shopping, people with no “opportunity passport” could only use small groceries and pharmacies, or else shop online. The protests that ensued did not cause the authorities to waver [25].

Since Poland, Lithuania’s neighbour, did not make such a move, and since its share of the adult population that had received at least a single vaccine dose used to be until July markedly similar to Lithuania’s, we wondered whether the introduction of the “opportunity passport” with its extended limitations of September 13 might have resulted from that moment on in better vaccination outcomes than those in Poland. Poland and Lithuania share very similar, relatively high levels (72% and 73% respectively) of trust in future EU’s decisions meant to combat the COVID-19 pandemic, and rather similar low distrust levels (24% vs. 27%). Regarding trust in the EU generally, with its 69%, Lithuania ranks third of all member states, while Poland ranks second (79%) in optimism about the future of the Union [20].

By contrast, the satisfaction of the residents of those countries with the measures taken by their own governments in dealing with the pandemic is in the medium EU range for Lithuania (56% satisfied and 44% not satisfied) and somewhat lower for Poland (53% satisfied, 42% not satisfied). Coupled with low trust in national governments, characteristic of many post-communist East and Central European countries, and deep distrust in the effectiveness of COVID-19 vaccines, this might jointly indicate low social cohesion and a low feeling of solidarity, which in its turn results in low levels of vaccination in those countries [26].

In view of the above, the aim of our study was to compare the effectiveness of vaccination programmes in two countries, Lithuania and Poland, measured by the respective percentages of the vaccinated population. The former state introduced a COVID-19 certificate domestically as a condition for participation in many everyday activities, while in the latter its possession did not entail any restrictions or benefits for the owner. Among the various possible strategies intended to lower infectivity, such as sero surveillance for COVID-19 antibodies, obligation to wear face masks in public, enforced social distancing or the use of COVID-19 certificates, we selected only the last one—the certificates with the accompanying restrictions.

## 2. Methods

### 2.1. Procedure and Analysed Data

Disaggregated vaccination data for Poland were taken from the Ministry of Health of the Republic of Poland and the Ministry of Health of the Republic of Lithuania, while the number of inhabitants in particular age brackets was taken from the accompanying European Centre for Disease Prevention (ECDC) data to ensure consistency. The measured period was four months—from 30 June 2021 to 31 October 2021—as in that period, the difference between Lithuania and Poland in rates of vaccination with at least a single dose rose from 1.47 p.p. to 13.81 p.p. The impact had to be measured from the perspective of actually achieving a uniformly high vaccination rate over all former LAU1 sub-regions (380 “powiats” in Poland and 60 “savivaldybės” in Lithuania) for the purpose of achieving herd immunity.

Additionally, data were analysed by age groups. For this purpose, we used ECDC data directly for age groups 10–14, 15–17, 18–24, 25–49 and 50–59. Unfortunately, from 1 September 2021 onward ECDC data begin to diverge from the data of the Ministry of Health of the Republic of Poland, as booster doses are counted as relating to new people receiving their first dose, which leads to serious discrepancy for those above 60. Lesser discrepancies should also affect other groups, as booster doses are also provided for healthcare workers and immunocompromised patients. Theoretically, the roll-out was at that moment covering people above 50, though in practice the waiting period of 6 months from the 2nd dose was mostly preventing big-scale vaccination with the 3rd dose of the 50–59 age bracket. To tackle this problem, additional data have been taken directly from the Ministry of Health of the Republic of Poland, though it provides them in differently divided age brackets, which allows comparison for age groups 60–69 and above 70. Such analyses allow us to gauge the effectiveness of the vaccine mandate in the most vulnerable age groups. Moreover, this allows us to gauge the indirect impact of the vaccine passport on those who are not compelled to vaccinate because of being under 16: whether it encourages them by creating a social norm or discourages them by turning vaccination into a tedious, best-avoided legal requirement.

### 2.2. Data Analysis Procedure

Vaccination increase of all analysed age brackets is presented in absolute and relative terms, and subsequently, the odds ratio (OR) for the existence of differences between the analysed countries has been calculated. The 95% confidence interval (95% CI) has been calculated to estimate the precision of the OR. A 5% level of significance was used for all hypothesis tests.

In order to determine to what extent mandates lead to a uniform increase of vaccination rates, we calculated the change in standard deviation (SD) of vaccination rates between former LAU1 sub-regions before and after the mandate. Additionally, as the key issue was how the least successful regions changed, the increase in the lowest quantile has been analysed. As prior studies were showing that prior track record was a very strong predictor of subsequent vaccination increase, a correlation between prior vaccination rates and the percentage of the remaining people that got vaccinated has been calculated using Spearman’s Rho with 95% confidence intervals, to be able to determine whether there has been any clear divergence. Maps were generated using GeoDa (Version 1.16.0.16), while statistical analysis was done in JASP (Version 0.15.0.0) and Dag-stat [27].

### 2.3. Outcome

The measured outcome is the divergence between Poland and Lithuania in the percentage of people who received at least a single dose of COVID-19 vaccine between 30 June 2021 and 31 October 2021, the time when in Lithuania COVID-19 vaccine mandates were first rolled out and later implemented. The divergence is measured both for age brackets and for former LAU1 sub-regions.

## 3. Results

**Figure 1:**
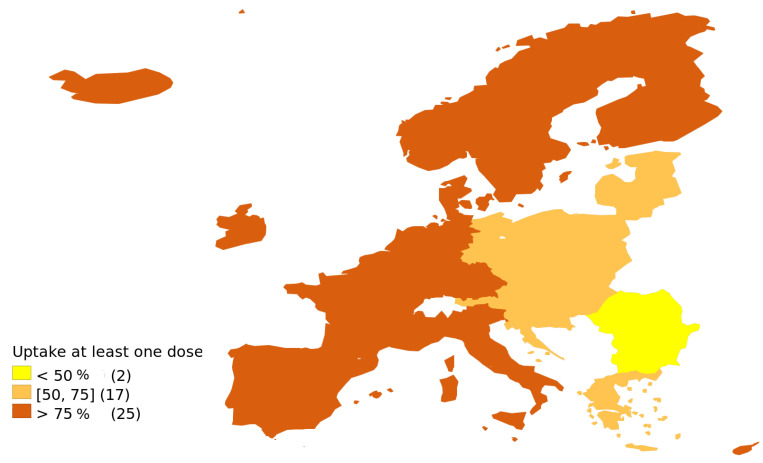
Vaccination rate of the European Economic Area—percentage of adults who received at least one dose of COVID-19 vaccine as of 30 September 2021, data for Germany presented in pre-unification borders, with Berlin excluded, based on Koch Institute [28]; source of data on other EEA states: ECDC [29].

**Figure 2:**
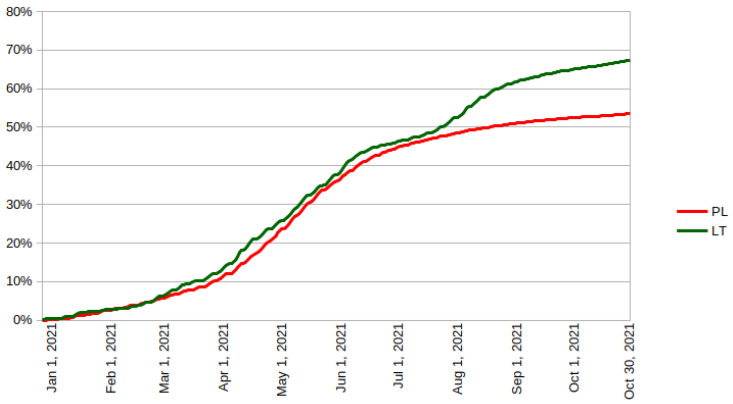
Share of the adult population that has received at least one dose of COVID-19 vaccine until a particular day, source of data: covidvax.live [30].

As presented in Figure 1, much of the EU has vaccination rates higher than 75%, with markedly lower rates in mostly former Eastern Bloc (such as Czechia, Slovakia, Croatia, Slovenia or the Baltic states), and the lowest in Bulgaria and Romania. When Germany is analysed in pre-unification borders, the gap between mostly former Eastern Bloc and the old EU becomes even more visible. Poland and Lithuania initially had very similar vaccination rates (cf. Figure 2), and until 26 July 2021, the difference remained within a 3 p.p. band. From that moment onward the trajectories clearly diverged, with the new peak of the number of people receiving the first vaccination dose reached in Lithuania on 4 August 2021, even though the highly restrictive mandate was formally announced only on 11 August 2021 and came into force on 13 September 2021, with further but much slower increasing divergence.

We analysed the increase in the percentages of those vaccinated by age groups in Poland and in Lithuania in the period June 30–October 31. In this period, Lithuania was clearly leading in the increase of vaccination rate, which was much higher than in the baseline scenario for Poland. Nevertheless, the net gain (calculated in each age group as the difference between gain for Lithuania and that for Poland) over the Polish baseline that could be attributed to the mandate clearly differed for various age brackets. The biggest success of Lithuania’s policy can be observed in the 15–17 age group, where the net gain was 16.5 p.p., even though the vaccine mandate in Lithuania actually concerned those at least 16 years old. In the 18–24 age bracket the net increase was 14.5 p.p., while among those aged 25–49 it was 13.8 p.p., and in the 50–59 age bracket—10.6 p.p. However, the net increase in the 60–69 group was 5.4 p.p. and only 3.0 p.p. for those over 70. Among the youngest, unaffected by the mandate, there has also been a net increase of 3.4 p.p., though the Polish level has not been reached.

Taking from these data only the number of those initially unvaccinated who received their first dose in the analysed period, one can clearly see that Lithuania was more successful in all age brackets, though its advantage was not uniform. In the 10–14 group, not covered by the mandate, the increase was 10.6% in Lithuania and 7.4% in Poland. In the partially covered by the mandate 15–17 age group, the increase was 35.5% and 13.5% respectively. In the 18–24 age group, it was respectively 43.1% and 11.6%, among those aged 25–49: 45.1% and 12.0%, among those aged 50–59: 41.3% and 18.2%, while in the 60–69 age bracket it was 40.2% and 18.2%. However, in the most vulnerable 70+ age group, even though Lithuania was more successful, it achieved vaccination of only 20.3% of those remaining, while for Poland, which did not introduce any restrictions, it was 18.9%. As analysed in Table 1, when OR was calculated for all age groups, both prior to and after vaccine mandate roll out, the differences were not only statistically significant but also confidence intervals were not overlapping, which shows that the divergence was a real phenomenon. Additionally, until June 30 in 3 age groups (10–14, 15–17 and 70+) Poland was in the lead, but after July 1 this trend was reversed.

**Figure 3:**
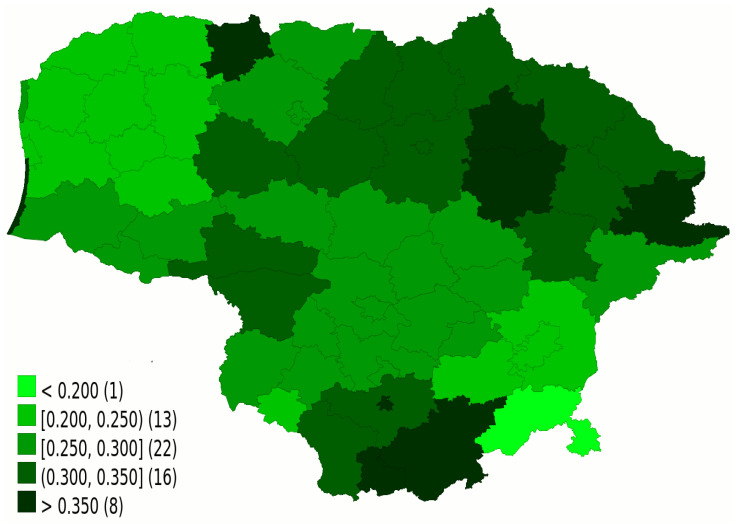
Vaccination rate increase between 30 June 2021 and 31 October 2021, as a percentage of the remaining population that got vaccinated. Source: Ministry of Health of the Republic of Lithuania.

As of 30 June 2021, the average percentage of the population that received at least a single dose of the vaccine for former Polish LAU1 sub-regions was 46.7% (SD 6.4%), while for Lithuania it was 45.0% (SD 8.4%). Subsequently, by 31 October 2021, this rate grew to 55.2% (SD 6.7%) for Poland and to 65.5% (SD 8.9%) for Lithuania. In Poland, the average rate for the least vaccinated quantile of sub-regions increased from 32.8% to 41.1%, while in Lithuania—from 36.0% to 55.6%. In both cases, the initial success of vaccination roll out in each sub-region was a strong predictor of the percentage of remaining people who would receive a vaccine in a subsequent period, which is presented in Figure 3. Spearman’s correlation between those two values for Polish sub-regions was 0.942 (CI 95% 0.929–0.952), while for Lithuanian ones—0.764 (CI 95% 0.632–0.852).

## 4. Discussion

Our results strongly support the thesis that a vaccine certificate that entails extensive everyday life restrictions is an effective motivator for individual decisions to vaccinate, and thus indirectly helps relieve the burden on the healthcare system. The mandate resulted in a 12.34 p.p. increase in the vaccination rate in Lithuania. As of 31 October 2021, Lithuania became the only post-communist country to have reached vaccination levels comparable with Austria and Greece, the states at the bottom of the ranking list of the old EU states. Vaccination hesitancy being a multi-conditioned phenomenon, it is not certain that a similar policy in a different country would have yielded analogous results.

From the perspective of achieving a uniformly high rate of vaccination among all sub-regions for the purpose of achieving herd immunity, the result was mixed, though. Regardless whether one measured the overall result change of standard deviation of former LAU1 sub-regions, or the change among the lowest quantile, there was no clear change, as the uniformity of the increase was comparable. In both cases, there was a very tiny increase in the divergence between sub-regions. Interestingly, mandates raised the percentage of vaccinated people comparably, both in earlier successful and unsuccessful regions. Though one could have hoped that they would especially raise laggers, this was not the case. It is possible that in the already well-vaccinated municipalities there was a higher number of facilities with restricted access that made the mandate more motivating, though minor divergence was observed in Poland as well. So, it could have been simply that in some districts there had been a higher number of people still considering vaccination.

Differences among children in the age group not subject to vaccine mandates were minor, and that group did not show any obvious signs of following a social norm or avoiding another burden imposed by the government. The mandate was the most effective in motivating the least vulnerable age brackets, i.e., teenagers and young adults, though there was some lesser positive impact up to age 69. Most worryingly, the impact on the most vulnerable age bracket of 70+ is at best minuscule.

The results are not totally surprising. Facilities requiring vaccine mandates (cinemas, restaurants, etc.) are concentrated mostly in cities and even normally are not frequented much by older people. Quite a uniform percentage of unvaccinated people deciding to get vaccinated would appear to suggest that it was simply the impact of restrictions and not some combination of factors where the mandate was merely the final straw. If we look at initial government assumptions, we could say that mandates were successful at achieving them, as in Lithuania vaccination rates reached 67.4% vis-à-vis 53.6% in Poland, so very near 70% that was theorised to be the level granting herd immunity, especially after including people who already recovered [31,32,33]. Here vaccine mandates, in spite of being blunt, clearly lead to increased vaccination rates among all age brackets below 70, so they brought some advantage for at least those moderately vulnerable.

One of the key factors in increasing the percentage of the vaccinated population in a country is effectively combating vaccine hesitancy, which, as studies show, is due to numerous factors, including social exclusion [34,35], age [36,37,38], gender [39,40,41] and education [42,43,44]. Interestingly, the percentage of those willing to get vaccinated even among healthcare professions (and students), excluding medicine proper, may not be markedly higher than among respondents unrelated to any medical profession or study, which might indicate a low potential of one’s professional or educational background to convince them to vaccinate [45]. As those studies, however, tend to be limited to narrow samples, rarely going beyond a single country, they have very limited ability to actually explain international differences in the willingness of citizens of a given state to get vaccinated.

Interestingly, however, even after three decades, the former Iron Curtain is still visible on the map of Europe (Figure 1) [3,46,47,48], with former eastern countries clearly underperforming in their ability to convince their population to receive the COVID-19 vaccine. This fact cannot be explained solely by economic factors, as according to Eurostat eastern European countries already have comparable or even higher GDP per capita in PPP than poorer Mediterranean countries. Neither can this be explained by education, as UN PISA tests show the north–south divide rather than the effect of the Iron Curtain. Among the post-communist countries, we noted the lowest vaccination coverage in Romania and Bulgaria (Figure 1), closely followed by Croatia, Slovakia, Poland, the former East Germany and Latvia, and by the somewhat higher levels of vaccination in the Czech Republic, Hungary, Estonia and Lithuania.

This rather consistent overall picture of low vaccination levels in East Europe might be due to a combination of factors, including not only the impact of anti-vaccine movements and their activity in social media (which is an obvious, general factor throughout Europe and beyond) but also inadequate organisational and even attitudinal response to the pandemic of top-level healthcare professionals in the countries concerned. A case in point is Bulgaria, the lowest-performing EU state in COVID-19 vaccinations, which seems to have experienced inconsistent activity in the early phase of the pandemic when very low numbers of those infected were coupled with very strict COVID-19 security measures, abruptly lifted in the summer of 2020. This in turn is likely to have led to a grave underestimation of the danger in the autumn of that year, which ultimately resulted in an overburdened healthcare system and in one of the highest COVID-19 morbidity rates in the EU; the low levels of vaccine acceptance were further aggravated by poor coordination of vaccine supply at the peak moment of the societal interest in getting vaccinated [49]. Similar factors and a similar scenario appear to have been decisive in the case of Romania [49], which, at the time of writing this, experiences the highest COVID-19 death toll in Europe, alongside Bulgaria and also Latvia, another poor vaccination outcome EU state [50]. By contrast, while the number of new (Delta variant) cases in Lithuania is soaring, its death rates, owing to relatively good vaccine uptakes due in turn to effective certificate-related incentives, are not as high. An additional factor in Bulgaria, Romania, Slovakia and Hungary might be the high share (7–10%) of the Roma population, characterised by low social inclusion, unsatisfactory access to healthcare and very low immunisation coverage levels even before the pandemic [51].

Differences between countries in vaccination outcomes may also be due to different timing of the first pandemic wave in individual countries and the death toll at that stage, as well as to different policies adopted in particular East European states both at the onset (e.g., the lockdown) and also in later stages of the pandemic, when vaccines were already available (such as the incentives rewarding the vaccinated segment of society). Unfortunately, the body of recent studies that analyse in depth COVID-19 vaccine hesitancy in those countries is not sufficient yet to allow meaningful cross-country comparisons. 

Similarly few studies exist to date on the effectiveness of various measures adopted by governments to increase vaccine uptake. In Germany, the effectiveness of three different strategies in combating vaccine hesitancy—providing freedoms, financial remuneration and vaccination at local doctors—was assessed by Klüver et al. [52]. All these factors seemed to play a role, especially effective if combined, with the freedoms most attractive to the younger generation, and the provision of the vaccination service locally—to the elderly. Patterns of COVID-19 vaccination in Israel and how they relate to various incentives were studied by Saban et al. [53], who found that those reluctant to vaccinate (especially young people) may be motivated by incentive schemes that impose restrictions, e.g., in access to social, cultural or sporting events, while a segment of society might also be encouraged by exemptions from quarantine.

In Lithuania, an opinion poll, made on a representative group of 1015 residents aged 18–75 between 25 January and 7 February 2021 by a market and public opinion research company Spinter research at the request of the Ministry of Health of the Republic of Lithuania [54], showed that jointly 64% of respondents would be willing to get vaccinated with an EU-approved vaccine, while 20% would rather or definitely not and 16% were undecided. Men, university graduates and older people were more willing to get vaccinated than women, people with lower education or younger respondents—in that respect Lithuania somewhat resembles Slovenia.

Another survey was conducted online on February 5–16 by NielsenIQ in Lithuania, Latvia and Estonia, with 1,600 respondents aged 16–64 in each country, representative with regard to age, gender and place of residence. Its results enabled the creation of a profile of a typical opponent of vaccination: aged 25–35, from a larger household, and from a lower income bracket. By contrast, a typical person expressing willingness to vaccinate was found to be aged 45–64, from a smaller household (2 members), with higher income and education level, and also more closely exposed to COVID-19 (e.g., because of the past illness of a family member). The group of vaccine sceptics was more likely to include service, trade workers, manual workers, the unemployed, or housewives; they were more likely to express more conservative views, were less receptive to innovation, and less likely to know personally someone with a COVID-19 infection. According to the survey, Latvians and Estonians are more sceptical about COVID-19 vaccination than Lithuanians: 34% of respondents in Latvia and 31% in Estonia declared that they would definitely not get vaccinated, whereas 51% of Latvians and the same ratio of Estonians declared the wish to do so [55].

At the end of August 2021, shortly before the extension of the application of the Lithuanian COVID-19 certificate, a survey commissioned by the local government of Vilnius was conducted among those not yet vaccinated to investigate the reasons for their decision [56]. Conducted by phone or the Internet, the survey included 1012 residents of Vilnius. The chief two reasons found for vaccine hesitancy were distrust of vaccines as developed too fast (22%) and support for various conspiracy theories (one-fourth of respondents—of these, 17% believe that vaccines are harmful but this information is withheld from the public, while 9% are convinced that the virus and vaccines are a major deception for humanity). Interestingly, one in ten respondents promised to get vaccinated later; another 7% claimed they still lacked information about the benefits of the vaccine. 

According to Kipras Krasauskas, vaccination coordinator in Vilnius, there were two essential insights from the survey, one highlighting the effectiveness of strict, “punitive” measures, and the other suggesting considering the use of various incentives and gifts in return for getting vaccinated—in other words, the stick-and-carrot motivation. The “stick” might be e.g., dismissal from work, a ban on freely travelling, or on participation in cultural events. That type of motivation seems to appeal most to the younger segment of the population. The “carrot” is mostly financial (a lottery with prizes, a voucher worth EUR 10–20, small gifts). However, as many as 47% said that their views on vaccination would not be changed by any incentive. Our own aggregate data on the number of those vaccinated do not allow us to identify individual reasons for the ultimate decision to vaccinate, beyond the differences among age groups or place of residence. Nevertheless, the payment of EUR 100 to each Lithuanian resident aged 75 or more, vaccinated with two doses between 1 September and 30 November 2021, or to those 75+ Lithuanians who have accepted the third, booster dose by 31 March 2022 [57], may have proved the effectiveness of such a financial reward in the oldest group, in which the increase between June 30 and September 31 was considerable, despite not reaching yet the Polish levels for that age bracket.

The Vilnius survey allowed the identification of four groups of those reluctant to get vaccinated [56]. In fact, only one of these groups—corresponding to “active resisters” [58]—might be deaf to incentives, while the other three groups—in various degrees corresponding to “the hesitant”, “the unconcerned” and “the poorly reached”—could probably be made to vaccinate with the right approach.

In Poland, COVID-19 vaccine hesitancy is quite high, the fact confirmed by our previous study [34]. Research conducted in April 2021, three months after the introduction in Poland of mass COVID-19 vaccination, on a representative adult sample [59], indicated that 30% of respondents were still unwilling to get vaccinated. 

Poland is also characterised by very low levels of acceptance of various restrictions for those without a vaccine passport. In an Ipsos survey conducted in March–April 2021 [60], only 36% of Poles (the second-lowest result among 28 studied countries worldwide) agreed that these passports should be required in shops, restaurants or offices, while only 49% (the third-lowest result) believed that they should be required at large public venues such as concert halls and stadiums.

A similar study conducted in mid-November 2021 by phone on a representative group [61] showed 54% of Poles supporting a ban on access to restaurants, cinemas and public events such as concerts, while 41% rather or strongly disagreed, with 5% undecided. Mandatory vaccinations for medical staff were supported by 65%, for teachers—by 58%, while employer’s access to information about an employee’s vaccination—by 56% of those surveyed. 

## 5. Limitations

There were some data quality issues concerning the vaccination rate. In the case of ECDC data, Polish booster doses were misclassified as a first dose, which—if taken at face value—would have led to a different conclusion. This forced us to crosscheck the data with local health ministries, and on this basis, we introduced corrections to our data before we made any calculations.

In addition, using Poland as a benchmark for what would happen in Lithuania without a vaccine mandate is a rough approximation, since there is clearly a different social dynamic in both countries, which was presented when data were disaggregated for age brackets. This commends caution in cases where the divergence is small.

Our study concentrates on objective measurable metrics indicating whether vaccine passports lead to the vaccination rate increase. It is beyond the scope of this work to analyse the ethical, legal or technical ramifications. Moreover, our success criteria were short-term vaccination rate increase and at the moment of writing this study, it is too early to answer how it affected the inevitable trade-offs, including especially the risk of increasing pandemic fatigue [62].

## 6. Conclusions

In the analysed period from 31 July to 30 September 2021, Lithuania passed and implemented a vaccine mandate. Simultaneously, the vaccination rate, measured as the number of people who received at least a single dose of the COVID-19 vaccine, grew markedly faster than in Poland, which relied only on public health campaigns and therefore could be used as a control group. The gap in vaccination outcomes between the two countries increased in the analysed period by 12.34 p.p. The increase was observed in all age groups that were subject to the mandate, though its impact was strongest in younger age brackets and very weak among people over 70. On the sub-regional level, a subsequent further divergence of local vaccination rates from the original values was observed, though this process was actually slightly weaker than in Poland.

## Figures and Tables

**Table 1:** Odds ratio to be vaccinated Lithuania vs. Poland.

Age (years)	10–14	15–17	18–24	25–49	50–59	60–69	Over 70
Period	Until 30 June 2021						
OR	0.555	0.732	1.42	1.329	1.026	1.249	0.505
95%CI	0.544–0.566	0.721–0.744	1.408–1.434	1.324–1.335	1.019–1.032	1.240–1.259	0.501–0.508
*p*	<0.001	<0.001	<0.001	<0.001	<0.001	<0.001	<0.001
Period	After 1 July 2021						
OR	1.482	3.529	5.770	6.006	4.346	3.032	1.089
95%CI	1.454–1.511	3.463–3.597	5.694–5.847	5.967–6.046	4.299–4.392	2.993–3.073	1.074–1.105
*p*	<0.001	<0.001	<0.001	<0.001	<0.001	<0.001	<0.001

## Data Availability

https://www.gov.pl/web/szczepimysie/mapa-punktow-szczepien; https://www.gov.pl/web/szczepienia-gmin/sprawdz-poziom-wyszczepienia-mieszkancow-gmin; https://www.gov.pl/web/szczepimysie/raport-szczepien-przeciwko-covid-19; https://covidvax.live/; https://www.rki.de/DE/Content/InfAZ/N/Neuartiges_Coronavirus/Daten/Impfquoten-Tab.html; https://vaccinetracker.ecdc.europa.eu/public/extensions/COVID-19/vaccine-tracker.html; https://koronastop.lrv.lt/en/#statistics-in-lithuania (accessed on 23 November 2021).

## Abbreviations

COVID-19: the coronavirus disease 2019, European Union: EU, percentage points: p.p.

## References

[1] Chwalba A., Zamorski K. (2020). The Polish-Lithuanian Commonwealth: History, Memory, Legacy.

[2] Butterwick R. (2021). The End of a Nation. Hist. Today.

[3] Fuchs-Schündeln N., Schündeln M. (2020). The Long-Term Effects of Communism in Eastern Europe. J. Econ. Perspect..

[4] Aburto J.M., Schöley J., Kashnitsky I., Zhang L., Rahal C., Missov T.I., Mills M.C., Dowd J.B., Kashyap R. (2021). Quantifying Impacts of the COVID-19 Pandemic through Life-Expectancy Losses: A Population-Level Study of 29 Countries. Int. J. Epidemiol..

[5] Islam N., Jdanov D.A., Shkolnikov V.M., Khunti K., Kawachi I., White M., Lewington S., Lacey B. (2021). Effects of Covid-19 Pandemic on Life Expectancy and Premature Mortality in 2020: Time Series Analysis in 37 Countries. BMJ.

[6] Henley J. (2021). Covid Passports: What Are European Countries Doing?. The Guardian.

[7] EU Digital COVID Certificate. https://ec.europa.eu/info/live-work-travel-eu/coronavirus-response/safe-covid-19-vaccines-europeans/eu-digital-covid-certificate_en.

[8] Giuffrida A. (2021). Italy Imposes ‘Green Pass’ Restrictions on Unvaccinated People. The Guardian.

[9] (2021). Covid: Italy to Require All Workers to Show “Green Pass” Certificate. BBC News.

[10] Niestadt M., Claros E. EPRS | European Parliamentary Research Service. Domestic Use of EU Digital Covid Certificates. PE 698.763—26 October 2021. https://www.europarl.europa.eu/RegData/etudes/BRIE/2021/698763/EPRS_BRI(2021)698763_EN.pdf.

[11] Paterlini M. (2021). Covid-19: Italy Sees Protests against Mandatory Health Passports for Workplaces. BMJ.

[12] Macron Gambled on Coronavirus Immunity Passport–and Won. https://www.politico.eu/article/macron-france-covid-immunity-passport-vaccine-pass/.

[13] COVID-19 Health Passes for Accessing Public Spaces Becoming the Norm in EU—16 Countries Implement Such Requirements. https://www.schengenvisainfo.com/news/covid-19-health-passes-for-accessing-public-spaces-becoming-the-norm-in-eu-16-countries-implement-such-requirements/.

[14] Measures to Contain the Spread of COVID-19 Infections | GOV.SI. https://www.gov.si/en/topics/coronavirus-disease-covid-19/measures-to-contain-the-spread-of-covid-19-infections/.

[15] Czech Republic Imposes New Restrictions as Infections Soar. https://news.yahoo.com/czech-republic-hit-rise-infections-090937099.html.

[16] Statistics | Eurostat. https://ec.europa.eu/eurostat/databrowser/view/NAMA_10_CO3_P3__custom_1620239/default/table?lang=en.

[17] COVID Pass Estonia: Estonia Vaccine Passport. https://www.covidpasscertificate.com/estonian-covid-certificate/.

[18] Saeima Adopts “No Covid Certificate, No Job” Law. https://eng.lsm.lv/article/economy/employment/latvian-saeima-adopts-no-covid-certificate-no-job-law.a428804/.

[19] Restrictions to Be Loosened for Covid Certificate Holders as of November 15. https://eng.lsm.lv/article/society/society/restrictions-to-be-loosened-for-covid-certificate-holders-as-of-november-15.a429466/.

[20] Standard Eurobarometer 95—Spring 2021—Wrzesień 2021—Eurobarometer Survey. https://europa.eu/eurobarometer/surveys/detail/2532.

[21] Galimybių Pasas šalies Viduje Atsirastų Anksčiau už Europinį. https://www.delfi.lt/a/86997961.

[22] Siūlo Naują Tvarką: Į Darbą—tik su Galimybių pasu. https://www.delfi.lt/a/87664763.

[23] BNS Daugiau Veiklų Reikės Galimybių Paso, Sako Premjerė. https://www.vz.lt/verslo-aplinka/2021/07/13/daugiau-veiklu-reikes-galimybiu-paso-sako-premjere.

[24] Ekonomikos ir Inovacijų Ministrė: Galimybių Pasas Gali Būti Naudojamas Vis Plačiau. https://www.lrt.lt/naujienos/verslas/4/1457746/ekonomikos-ir-inovaciju-ministre-galimybiu-pasas-gali-buti-naudojamas-vis-placiau.

[25] BNS U.A. Protestas Prieš Galimybių Pasą Vilniuje Baigėsi Anksčiau Nei Planuota, Dalyvavo Apie 100 žmonių. https://www.15min.lt/naujiena/aktualu/lietuva/vilniuje-vyksta-protesto-akcija-pries-ribojimus-galimybiu-paso-neturintiems-gyventojams-56-1567236.

[26] Trust Deficit Stalls Vaccinations in Eastern Europe, Driving New COVID Surge. https://www.politico.eu/article/covid-vaccination-eastern-europe-trust-fourth-wave-vaccine/.

[27] Mackinnon A. (2000). A Spreadsheet for the Calculation of Comprehensive Statistics for the Assessment of Diagnostic Tests and Inter-Rater Agreement. Comput. Biol. Med..

[28] RKI—Coronavirus SARS-CoV-2—Digitales Impfquotenmonitoring Zur COVID-19-Impfung. https://www.rki.de/DE/Content/InfAZ/N/Neuartiges_Coronavirus/Daten/Impfquoten-Tab.html.

[29] COVID-19 Vaccine Tracker | European Centre for Disease Prevention and Control. https://vaccinetracker.ecdc.europa.eu/public/extensions/COVID-19/vaccine-tracker.html#uptake-tab.

[30] Covidvax.Live: Live COVID-19 Vaccination Tracker—See Vaccinations in Real Time!. http://covidvax.live/.

[31] Feleszko W., Lewulis P., Czarnecki A., Waszkiewicz P. (2021). Flattening the Curve of COVID-19 Vaccine Rejection—An International Overview. Vaccines.

[32] Jung F., Krieger V., Hufert F.T., Küpper J.-H. (2020). Herd Immunity or Suppression Strategy to Combat COVID-19. Clin. Hemorheol Microcirc.

[33] Sanche S., Lin Y.T., Xu C., Romero-Severson E., Hengartner N., Ke R. (2020). High Contagiousness and Rapid Spread of Severe Acute Respiratory Syndrome Coronavirus 2. Emerg Infect. Dis.

[34] Walkowiak M.P., Walkowiak D. (2021). Predictors of COVID-19 Vaccination Campaign Success: Lessons Learnt from the Pandemic So Far. A Case Study from Poland. Vaccines.

[35] Malik A.A., McFadden S.M., Elharake J., Omer S.B. (2020). Determinants of COVID-19 Vaccine Acceptance in the US. EClinicalMedicine.

[36] Domnich A., Cambiaggi M., Vasco A., Maraniello L., Ansaldi F., Baldo V., Bonanni P., Calabrò G.E., Costantino C., de Waure C. (2020). Attitudes and Beliefs on Influenza Vaccination during the COVID-19 Pandemic: Results from a Representative Italian Survey. Vaccines.

[37] Soares P., Rocha J.V., Moniz M., Gama A., Laires P.A., Pedro A.R., Dias S., Leite A., Nunes C. (2021). Factors Associated with COVID-19 Vaccine Hesitancy. Vaccines.

[38] Kourlaba G., Kourkouni E., Maistreli S., Tsopela C.-G., Molocha N.-M., Triantafyllou C., Koniordou M., Kopsidas I., Chorianopoulou E., Maroudi-Manta S. (2021). Willingness of Greek General Population to Get a COVID-19 Vaccine. Glob. Health Res. Policy.

[39] Sowa P., Kiszkiel Ł., Laskowski P.P., Alimowski M., Szczerbiński Ł., Paniczko M., Moniuszko-Malinowska A., Kamiński K. (2021). COVID-19 Vaccine Hesitancy in Poland-Multifactorial Impact Trajectories. Vaccines.

[40] Schwarzinger M., Watson V., Arwidson P., Alla F., Luchini S. (2021). COVID-19 Vaccine Hesitancy in a Representative Working-Age Population in France: A Survey Experiment Based on Vaccine Characteristics. Lancet Public Health.

[41] Edwards B., Biddle N., Gray M., Sollis K. (2021). COVID-19 Vaccine Hesitancy and Resistance: Correlates in a Nationally Representative Longitudinal Survey of the Australian Population. PLoS ONE.

[42] Afifi T.O., Salmon S., Taillieu T., Stewart-Tufescu A., Fortier J., Driedger S.M. (2021). Older Adolescents and Young Adults Willingness to Receive the COVID-19 Vaccine: Implications for Informing Public Health Strategies. Vaccine.

[43] Bono S.A., Faria de Moura Villela E., Siau C.S., Chen W.S., Pengpid S., Hasan M.T., Sessou P., Ditekemena J.D., Amodan B.O., Hosseinipour M.C. (2021). Factors Affecting COVID-19 Vaccine Acceptance: An International Survey among Low- and Middle-Income Countries. Vaccines.

[44] Dodd R.H., Cvejic E., Bonner C., Pickles K., McCaffery K.J. (2021). Sydney Health Literacy Lab COVID-19 group Willingness to Vaccinate against COVID-19 in Australia. Lancet Infect. Dis.

[45] Petravić L., Arh R., Gabrovec T., Jazbec L., Rupčić N., Starešinič N., Zorman L., Pretnar A., Srakar A., Zwitter M. (2021). Factors Affecting Attitudes towards COVID-19 Vaccination: An Online Survey in Slovenia. Vaccines.

[46] Grzymala-Busse A. (2016). An East-West Split in the EU?. Curr. Hist..

[47] Simko V., Ginter E. (2013). Impact of Political Systems on European Population Health. J. Pol. Sci. Pub. Aff.

[48] Safaei J. (2012). Post-Communist Health Transitions in Central and Eastern Europe. Econ. Res. Int..

[49] Boytchev H. (2021). Covid-19: Why the Balkans’ Vaccine Rollout Lags behind Most of Europe. BMJ.

[50] *The Economist*. 13 November 2021. https://www.economist.com/europe/2021/11/13/eastern-european-countries-are-being-hit-by-a-wave-of-covid-deaths.

[51] Roma. https://minorityrights.org/minorities/roma-16/.

[52] Klüver H., Hartmann F., Humphreys M., Geissler F., Giesecke J. (2021). Incentives Can Spur COVID-19 Vaccination Uptake. Proc. Natl. Acad. Sci. USA.

[53] Saban M., Myers V., Ben Shetrit S., Wilf-Miron R. (2021). Issues Surrounding Incentives and Penalties for COVID-19 Vaccination: The Israeli Experience. Prev. Med..

[54] Tyrimas Parodė, Kiek Lietuvos Gyventojų Sutiktų Skiepytis Nuo Koronaviruso. https://spinter.lt/site/lt/vidinis_noslide/menutop/9/home/publish/MTQ1ODs5Ozsw.

[55] BNS Nenorinčiųjų Skiepytis Demografinis Portretas Neramina Sociologus. https://www.vz.lt/verslo-aplinka/2021/03/08/nenorinciuju-skiepytis-demografinis-portretas-neramina-sociologus.

[56] Tyrimas: Vilnius Nustatė Skiepų Priešininko Portretą. https://www.lrt.lt/naujienos/sveikata/682/1509079/tyrimas-vilnius-nustate-skiepu-priesininko-portreta.

[57] Išmokos Senjorams, Pasiskiepijusiems nuo COVID-19: Dažniausiai Užduodami Klausimai. https://socmin.lrv.lt/lt/naujienos/ismokos-senjorams-pasiskiepijusiems-nuo-covid-19-dazniausiai-uzduodami-klausimai.

[58] Let’s Talk about Protection: Enhancing Childhood Vaccination Uptake. https://www.ecdc.europa.eu/en/publications-data/lets-talk-about-protection-enhancing-childhood-vaccination-uptake.

[59] Raciborski F., Samel-Kowalik P., Gujski M., Pinkas J., Arcimowicz M., Jankowski M. (2021). Factors Associated with a Lack of Willingness to Vaccinate against COVID-19 in Poland: A 2021 Nationwide Cross-Sectional Survey. Vaccines.

[60] Ipsos-WEF-Global Views on Personal Health Data and Vaccine Passports—Graphic Report—2021-04-26.Pdf. https://www.ipsos.com/sites/default/files/ct/news/documents/2021-04/Ipsos-WEF%20-Global%20Views%20on%20Personal%20Health%20Data%20and%20Vaccine%20Passports%20-%20Graphic%20Report%20-%202021-04-26.pdf.

[61] Obostrzenia dla niezaszczepionych? Nowy sondaż “Wydarzeń” Polsatu—Polsat News. https://www.polsatnews.pl/wiadomosc/2021-11-19/obowiazkowe-szczepienia-i-obostrzenia-wobec-niezaszczepionych-nowy-sondaz-dla-wydarzen-polsatu/.

[62] WHO-EURO-2020-1160-40906-55390-Eng.Pdf. https://apps.who.int/iris/bitstream/handle/10665/335820/WHO-EURO-2020-1160-40906-55390-eng.pdf.

